# FOG Random Drift Signal Denoising Based on the Improved AR Model and Modified Sage-Husa Adaptive Kalman Filter

**DOI:** 10.3390/s16071073

**Published:** 2016-07-12

**Authors:** Jin Sun, Xiaosu Xu, Yiting Liu, Tao Zhang, Yao Li

**Affiliations:** Key Laboratory of Micro-Inertial Instrument and Advanced Navigation Technology, Ministry of Education, School of Instrument Science and Engineering, Southeast University, Nanjing 210096, China; sunjin8607986@126.com (J.S.); gcdlyt1985@163.com (Y.L.); ztandyy@163.com (T.Z.); lyjenny11@163.com (Y.L.)

**Keywords:** fiber optic gyroscope (FOG), auto regressive (AR) model, Sage-Husa adaptive Kalman filter (SHAKF), online model, random drift

## Abstract

In order to reduce the influence of fiber optic gyroscope (FOG) random drift error on inertial navigation systems, an improved auto regressive (AR) model is put forward in this paper. First, based on real-time observations at each restart of the gyroscope, the model of FOG random drift can be established online. In the improved AR model, the FOG measured signal is employed instead of the zero mean signals. Then, the modified Sage-Husa adaptive Kalman filter (SHAKF) is introduced, which can directly carry out real-time filtering on the FOG signals. Finally, static and dynamic experiments are done to verify the effectiveness. The filtering results are analyzed with Allan variance. The analysis results show that the improved AR model has high fitting accuracy and strong adaptability, and the minimum fitting accuracy of single noise is 93.2%. Based on the improved AR(3) model, the denoising method of SHAKF is more effective than traditional methods, and its effect is better than 30%. The random drift error of FOG is reduced effectively, and the precision of the FOG is improved.

## 1. Introduction

With the development of fiber optical technology, the fiber optic gyroscope (FOG) is gradually replacing other types of gyroscopes with its unique advantages, and it has become mainstream in inertial navigation system applications [1]. At present, much research work in modeling FOG random drift is being carried out at home and abroad. When the initial alignment of a strapdown inertial navigation system (SINS), the core of which is a FOG, is carried out, FOG random drift is an important factor that influences the alignment precision [2]. Through modeling and filtering, the effect of FOG random drift can be effectively restrained. The traditional modeling of FOG random drift is limited to an offline form, which uses a random drift model based on the output data of a single gyroscope obtained in the laboratory. Additionally, the estimated model is of certain significance in analyzing the characteristic of random drift due to changeable measurement conditions under the circumstances of the moment, such as temperature, humidity, electromagnetic field and gravity, as well as the impact caused by restarting the gyroscope; the reliability of a model established offline will decrease, and its universality is not strong [3,4]. To solve the above problems, we need to develop a real-time filtering method of online modeling of FOG random drift, based on real-time observations at each restart of the gyroscope, so an online model of FOG random drift can be established with a software method, and the model then is used to realize real-time filtering on the FOG.

In [1,2,3,4,5], auto regressive moving average (ARMA) models are used to establish the random drift error model of FOG and a ring laser gyroscope (RLG); the Kalman filter is adopted to filter the zero the drift of FOG and RLG, and the Allan variance analysis method is used to analyze various data noise sources before and after modeling and filtering. The ARMA(*n*, *m*) models require a stable signal, with a normal distribution and zero mean time series, which is also necessary for modeling gyroscope signals. Therefore, the modeling and filtering of gyroscope signals is performed only with post-processing. The estimated noise source error coefficient or performance parameters of the FOG are not suitable for inertial navigation systems used in practical applications.

In [6], an improved AR(2) model is proposed to establish the random drift error model of FOG online, and then, the Kalman filtering algorithm is adopted to filter the random drift error of FOG. Jin [7] proposed an online modeling and filtering method. In this method, the traditional offline model was improved based on a large number of measured data. In addition, a method of the random drift model of FOG based on the AR model is studied, then an $H_{\infty }$ filter is designed for filtering the signal online. An improved AR model of FOG random drift error and a forward linear prediction (FLP) filter were designed by Wang [8]. In [9], adaptive moving average (AMA) and random weighting estimation (RWE) based on double-factor adaptive KF algorithm, named AMA-RWE-DFAKF was proposed to denoise FOG drift signals under both static and dynamic conditions. Han [10] undertook research on the wavelet filtering method of FOG output signals. It is based on the combination of a Mallat pyramid algorithm and the characteristics of a finite impulse response (FIR) filter. Additionally, an equivalent FIR filtering algorithm was deduced based on wavelets. On the basis of the wavelet threshold filtering, a real-time wavelet filtering method for FOG output signals was given. In [11], FOG state estimation was combined with an autoregressive integrated moving average (ARIMA) model for non-linear parameter estimation. A Gaussian particle filter (GPF) was used to achieve ARIMA model identification and state estimation of a FOG.

In this paper, an improved AR(3) model is suggested, where the FOG random drift model is established online using measured FOG signal instead of a signal with zero mean. After modeling of real-time data at each restart of a gyroscope with the improved AR(3) model, direct filtering of the FOG signal is conducted using SHAKF. Filtering results are analyzed with Allan variance.

The rest of this paper is organized as follows: the online model of FOG random drift based on the improved AR model is introduced in Section 2. The modified SHAKF algorithm is described in Section 3. In Section 4, the practical implementation of the proposed method is introduced. The results are given for static and dynamic experiments to verify the feasibility of the improved AR model and modified SHAKF. Finally, conclusions are drawn in Section 5.

## 2. Online Modeling of FOG Random Drift

Time series analysis methods are commonly used to model the random drift error of FOG. ARMA modeling is a time series analysis method for analyzing observed random data. ARMA modeling involves fitting the suitable ARMA(*n*, *m*) model to the observed time series $\left\{x_{t},t=1,2,\ldots ,M\right\}$. The estimation process of ARMA model parameters is a nonlinear regression process, and its calculation is very complex. Enough high order AR models can be used to replace ARMA models to avoid complexity in the parameter estimation of the ARMA model. The AR model is a class of ARMA model, because its parameter estimation is a linear estimation, and the calculation is simple and fast. As such, the model has great advantages in engineering applications.

By adopting the method proposed in [1] for offline analysis on a certain type of FOG static data under different conditions, it is shown that the AR model can better fit FOG random drift, so in this paper, an improved AR model is used to realize the modeling of gyroscope random drift time series, estimate model parameters in real time and achieve online modeling.

Gyroscope output needs to meet the three conditions of being stable with a normal distribution and zero mean when the AR model is used to model gyroscope drift time series [2]. The analysis results of a certain type of FOG static output data under measurement conditions show that, gyroscope static data always satisfy the conditions of stationarity and normality; but, they do not meet the condition of zero mean, and the detected means are different after each restarting. The cause of this phenomenon is as follows: gyroscopes can measure the Earth’s rotation rate of a geographical location. Due to the impact of current surges, electromagnetic interference and other factors, the mean of the gyroscope will drift in a small range after each restarting. The value is steady (constant) after the stabilization of a gyroscope. The constant after stabilization contains the Earth’s rotation rate and (smaller) constant drift measured by the gyroscope. Therefore, the zero mean problem should be solved when adopting an AR model for online modeling. In continuation, an improved AR model is proposed as a solution of the described problem.

### 2.1. The Principle of Online Modeling

The framework of the AR(*n*) model is:

(1) $$x_{k}=\varphi_{1}x_{k-1}+\varphi_{2}x_{k-2}+\ldots +\varphi_{n}x_{k-n}+a_{k}$$

where *k* is the sequence number, and its range is [1,+∞]; *n* is the order of AR, and its range is n≥1, and *k*, *n* are integers; $x_{k}$ is the observed time series; $\varphi_{1}\sim \varphi_{n}$ are parameters to be estimated; $a_{k}$ is white noise. On the assumption that the collected gyroscope data $z_{1},z_{2}\ldots z_{k},z_{k+1}\ldots$ are stationary and a normal sequence, the AR model can be established by the traditional method with a zero mean value. The observed time series can be written using the mean value of the sequence $\bar{z}$ as follows:

(2) $$\{\begin{array}{c} x_{k}=z_{k}-\bar{z}\\ x_{k-1}=z_{k-1}-\bar{z}\\ x_{k-2}=z_{k-2}-\bar{z}\\ \cdots \\ x_{k-n}=z_{k-n}-\bar{z} \end{array}$$

By writing Equation (2) into Equation (1), the following expression is obtained:

(3) $$z_{k}=\varphi_{1}z_{k-1}+\varphi_{2}z_{k-2}+\ldots +\varphi_{n}z_{k-n}+(1-\varphi_{1}-\varphi_{2}\ldots -\varphi_{n})\cdot \bar{z}+a_{k}$$

From the above theoretical analysis, we can know that the average value $\bar{z}$ of the FOG static output data should be constant after gyroscope stabilization after restarting. After the model is established, $\varphi_{1}\sim \varphi_{n}$ are also constant, so we denote $c=(1-\varphi_{1}-\varphi_{2}-\ldots -\varphi_{n})\cdot \bar{z}$. Equation (3) is now rewritten as:

(4) $$z_{k}=\varphi_{1}z_{k-1}+\varphi_{2}z_{k-2}+\ldots +\varphi_{n}z_{k-n}+c+a_{k}$$

and describes the dynamic AR(*n*) model. The recursive least squares (RLS) method is used to estimate unknown parameters in real time.

### 2.2. Estimation of Model Parameters

The estimation methods of AR model parameters can be divided into two categories: direct estimation methods and recursive estimation methods. The former use observed data or the statistical properties directly to estimate the model parameters, among which the least squares complex exponential (LSCE) method is a most popular method. Due to how much data are required to estimate the parameters accurately being unknown, these kinds of methods select the length of the time window empirically. When the length is too small, this will make the parameters inaccurate; however, when an excessive length is applied, the time demanded makes these methods only be able to be used in offline modeling. The recursive estimation methods can estimate the model parameters in real time, and the RLS method is selected to estimate the parameters of the FOG random drift model in this paper.

Substituting the collected output $\left\{z_{k},k=1,2,\ldots ,N\right\}$ at the initial time into Equation (4), then the following linear equations can be obtained [12,13]:

(5) $$\{\begin{array}{c} z_{n+1}=\varphi_{1}z_{n}+\varphi_{2}z_{n-1}+\ldots +\varphi_{n}z_{1}+c+a_{n+1}\\ z_{n+2}=\varphi_{1}z_{n+1}+\varphi_{2}z_{n}+\ldots +\varphi_{n}z_{2}+c+a_{n+2}\\ \cdots \\ z_{N}=\varphi_{1}z_{N-1}+\varphi_{2}z_{N-2}+\ldots +\varphi_{n}z_{N-n}+c+a_{N} \end{array}$$

where *n* is the order of AR.

The above equation can be written in the form:

(6) $$Y_{N}=Z_{N}\theta_{N}+a_{N}$$

where $Y_{N}={\left[\begin{array}{cccc} z_{n+1} & z_{n+2} & \cdots & z_{N} \end{array}\right]}_{1\times (N-n)}^{T}$, $\theta_{N}={\left[\begin{array}{ccccc} \varphi_{1} & \varphi_{2} & \cdots & \varphi_{n} & c \end{array}\right]}_{1\times (n+1)}^{T}$, $a_{N}={\left[\begin{array}{cccc} a_{n+1} & a_{n+2} & \cdots & a_{N} \end{array}\right]}_{1\times (N-n)}^{T}$, $Z_{N}={\left[\begin{array}{ccccc} z_{n} & z_{n-1} & \cdots & z_{1} & 1\\ z_{n+1} & z_{n} & \cdots & z_{2} & 1\\ \cdots \\ z_{N-1} & z_{N-2} & \cdots & z_{N-n} & 1 \end{array}\right]}_{\left(N-n)\times (n+1\right)}$.

According to the theory of multiple regression, the least square estimation of the parameter matrix ***θ****_N_* is:

(7) $$\theta_{N}={\left(Z_{N}^{T}Z_{N}\right)}^{-1}Z_{N}^{T}Y_{N}$$

Assuming that the model parameter of the observed sequence at the present stage is $\theta_{N}$ and with the arrival of new data $z_{N+1}$, an updated estimation of $\theta_{N}$ can be obtained based on sequence $\left\{z_{t},t=1,2,\ldots ,N,N+1\right\}$. According to the form of the above matrix, the least square estimation based on N + 1 data is as follows:

(8) $$\theta_{N+1}=p_{N+1}Z_{N+1}^{T}Y_{N+1},\;p_{N+1}={\left(Z_{N+1}^{T}Z_{N+1}\right)}^{-1}$$

where *h* is sequence number, and its range is [1,+∞]; *h* is an integer. $Z_{N+1}={\left[\begin{array}{c} Z_{N}\\ Z_{\left(h+1\right)} \end{array}\right]}_{\left(N-n+1)\times (n+1\right)}$, $Y_{N+1}={\left[\begin{array}{c} Y_{N}\\ z_{N+1} \end{array}\right]}_{(N-n+1)\times 1}$, $Z_{\left(h+1\right)}={\left[\begin{array}{ccccc} z_{N} & z_{N-1} & \cdots & z_{N-n+1} & 1 \end{array}\right]}_{1\times (n+1)}$.

According to the multiplication of the block matrix, the following expression can be obtained:

(9) $$\{\begin{array}{c} Z_{N+1}^{T}Y_{N+1}=Z_{N}^{T}Y_{N}+Z_{\left(h+1\right)}^{T}z_{N+1}\\ p_{N+1}={\left(p_{N}^{-1}+Z_{\left(h+1\right)}^{T}Z_{\left(h+1\right)}\right)}^{-1} \end{array}$$

By the matrix inversion formula, we can get the following:

(10) $$p_{N+1}=(I+p_{N}\frac{Z_{\left(h+1\right)}^{T}Z_{\left(h+1\right)}}{1+Z_{\left(h+1\right)}p_{N}Z_{\left(h+1\right)}^{T}})p_{N}$$

where $I_{\left(n+1)\times (n+1\right)}$ is an identity matrix.

Substituting Equations (9) and (10) into Equation (8), the recursive estimation formula of the parameters is obtained:

(11) $$\theta_{N+1}=\theta_{N}+K_{N+1}(z_{N+1}-Z_{\left(h+1\right)}\theta_{N})$$

where $K_{N+1}=\frac{1}{1+Z_{\left(h+1\right)}p_{N}Z_{\left(h+1\right)}^{T}}p_{N}Z_{\left(h+1\right)}^{T}$.

The above formula shows that the new estimation ***θ****_N+_*_1_ of the AR model parameters involves amending the original estimation ***θ****_N_*, and the correction term is $K_{N+1}(z_{N+1}-Z_{\left(h+1\right)}\theta_{N})$.

## 3. Real-Time Filtering

### 3.1. Selection of the Filter

**Figure 1:**
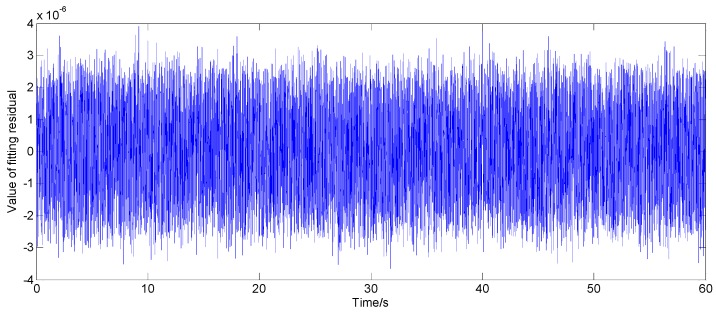
Fitting residual error sequence.

**Figure 2:**
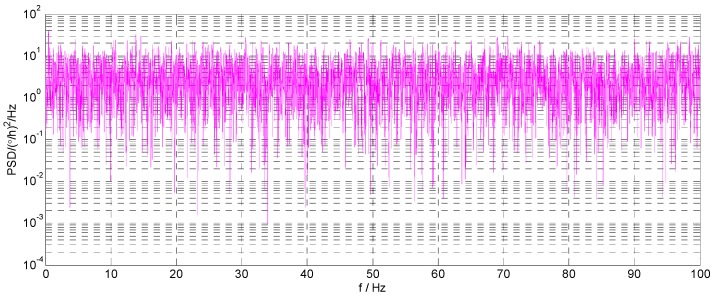
The PSD of the fitting residual error sequence.

After the AR model of FOG random drift is obtained, the traditional approach is to filter it through a Kalman filter [14,15,16,17,18,19]. The state space that sets the gyroscope as the research object is described as follows:

(12) $$\{\begin{array}{c} X_{t}=\Phi_{t,t-1}X_{t-1}+e_{t}\\ Z_{t}=H_{t}X_{t}+\varepsilon_{t} \end{array}$$

where $X_{t}$ is the state vector at time *t*; $\Phi_{t,t-1}$ is the state transition matrix; $e_{t}$ is the system process noise sequence; $Z_{t}$ is the observation vector at time *t*, and here, it is the gyroscope output; $H_{t}$ is the observation matrix; $\varepsilon_{t}$ is the measurement noise sequence; here, it is the fitting residual sequence of model. The state equation is the improved AR model of the gyroscope. Using a Kalman filter to filter the output signal of the gyroscope, the residual sequence $\varepsilon_{t}$ is generally regarded as white noise, but this approach is not reasonable. An improved AR(3) model is established by using the static output data of a certain type of FOG. The residual error sequence fitted by the model is shown in Figure 1, and the corresponding power spectral density (PSD) is shown in Figure 2.

According to [20], the PSD of white noise should be constant, and the power spectrum is equal to the variance intensity. As can be seen from Figure 2, the PSD of the residual series $\varepsilon_{t}$ is not constant, so it is not reasonable to simply treat the fitting residual part as white noise.

### 3.2. Sage-Husa Adaptive Kalman Filter

SHAKF, proposed by Sage and Husa [21], is an adaptive filtering algorithm for the uncertainty of noise statistical characteristics. A time varying noise estimator is added into the KF framework, which can estimate the statistical characteristics of noise in real time and mitigate the filter divergence.

#### 3.2.1. Design of SHAKF

Consider the stochastic linear discrete system [22,23]:

(13) $$\left\{ \begin{array}{l} X_{k}=\Phi_{k,k-1}X_{k-1}+W_{k-1}^{\prime }\\ Z_{k}=H_{k}X_{k}+V_{k}^{\prime } \end{array}\right.$$

where $X_{k}$ is the state vector at epoch *k*; $\Phi_{k,k-1}$ is the state transition matrix; $W_{k-1}^{\prime }$ is the system process noise sequence; $Z_{k}$ is the observation vector at epoch *k*; $H_{k}$ is the observation matrix; $V_{k}^{\prime }$ is the measurement noise sequence; both are Gauss white noise, which are independent of each other with time varying mean and covariance matrix; they meet the following conditions:

(14) $$\left\{ \begin{array}{l} E\left[W_{k}^{\prime }\right]=q_{k}\begin{array}{ccc} & & E\left[\left(W_{k}^{\prime }-q_{k}\right)\left(W_{k}^{\prime T}-q_{j}\right)\right]=Q_{k}\delta_{kj} \end{array}\\ E\left[V_{k}^{\prime }\right]=r_{k}\begin{array}{ccc} & & \begin{array}{c} E\left[\left(V_{k}^{\prime }-r_{k}\right)\left(V_{k}^{\prime T}-r_{k}\right)\right]=R_{k}\delta_{kj} \end{array} \end{array}\\ E\left[\left(W_{k}^{\prime }-q_{k}\right)\left(V_{k}^{\prime T}-r_{k}\right)\right]=0 \end{array}\right.$$

where $\delta_{kj}$ represents the Dirac delta function, $\delta_{kj}=\{\begin{array}{cc} 1, & if\;k=l\\ 0, & otherwise \end{array}$; $Q_{k}$ is the process noise covariance matrix; and $R_{k}$ is the measurement noise covariance matrix.

Because the process noise and measurement noise have a non-zero mean value, the system cannot directly use the Kalman filter. Now, we will derive the SHAKF algorithm briefly [22].

Equation (13) is rewritten as:

(15) $$\left\{ \begin{array}{l} X_{k}=\Phi_{k,k-1}X_{k-1}+q_{k-1}+W_{k-1}\\ Z_{k}=H_{k}X_{k}+r_{k}+V_{k} \end{array}\right.$$

where $W_{k-1}$ and $V_{k}$ are both white noise with zero mean.

According to Equation (15), the following equation can be obtained:

(16) $$\left\{ \begin{array}{l} {\hat{X}}_{k,k-1}=\Phi_{k,k-1}{\hat{X}}_{k-1}+q_{k-1}\\ \nu_{k}=Z_{k}-H_{k}{\hat{X}}_{k,k-1}-r_{k}\\ {\hat{X}}_{k}={\hat{X}}_{k,k-1}+K_{k}\nu_{k} \end{array}\right.$$

where ${\hat{X}}_{k,k-1}$ is the predicted state vector; ${\hat{X}}_{k}$ is the state estimation vector; $\nu_{k}$ is the residual error series vector; $K_{k}$ is the filtering gain.

Subtracting Equations (15) and (16), then we can get:

(17) $$X_{k}-{\hat{X}}_{k}=\Phi_{k,k-1}\left(X_{k-1}-{\hat{X}}_{k-1}\right)-K_{k}\nu_{k}+W_{k-1}$$

(18) $$\nu_{k}=H_{k}\left(X_{k}-{\hat{X}}_{k,k-1}\right)+V_{k}$$

Firstly, we will derive the estimation formula of the statistical characteristics of the measurement noise. Transposing both sides of Equation (18), the following can be obtained:

(19) $$\nu_{k}^{T}={\left(X_{k}-{\hat{X}}_{k,k-1}\right)}^{T}H_{k}^{T}+V_{k}^{T}$$

Furthermore, considering that the measurement noise and the estimation error are not related, we can get:

(20) $$E\left[\nu_{k}\nu_{k}^{T}\right]=E\left[H_{k}\left(X_{k}-{\hat{X}}_{k,k-1}\right){\left(X_{k}-{\hat{X}}_{k,k-1}\right)}^{T}H_{k}^{T}\right]+E\left[V_{k}V_{k}^{T}\right]$$

The above formula can be simplified as:

(21) $$E\left[\nu_{k}\nu_{k}^{T}\right]=H_{k}P_{k,k-1}H_{k}^{T}+R_{k}$$

where $P_{k,k-1}$ is the covariance matrix of the predicted state vector.

The formula is written in the form of a recursive estimation formula as:

(22) $${\hat{R}}_{k}=(1-d_{k}){\hat{R}}_{k-1}+d_{k}\left[\nu_{k}\nu_{k}^{T}-H_{k}P_{k,k-1}H_{k}^{T}\right]$$

where $d_{k}$ is a correction factor with $d_{k}=\frac{1-b}{1-b^{k+1}}$ and b is the forgetting factor, 0<b<1. Equation (22) is namely the recursive estimation formula of measurement noise ${\hat{R}}_{k}$ of the SHAKF algorithm.

Then, we derive the estimation formula of the statistical properties of process noise. Transposing both sides of Equation (17), the following can be obtained:

(23) $${\left(X_{k}-{\hat{X}}_{k}\right)}^{T}+{\nu_{k}}^{T}{K_{k}}^{T}={\left(X_{k-1}-{\hat{X}}_{k-1}\right)}^{T}{\Phi_{k,k-1}}^{T}+{W_{k-1}}^{T}$$

Both sides of Equation (17) are respectively multiplied by both sides of Equation (23), and then, the mathematical expectation is taken. Furthermore, considering that the process noise and the estimation error are not related, the estimated residual error is uncorrelated with the estimation error, and the process noise and the estimated residual error have zero expectation, so we can get:

(24) $$\begin{array}{l} E\left[W_{k-1}{W_{k-1}}^{T}\right]+\Phi_{k,k-1}E\left[\left(X_{k-1}-{\hat{X}}_{k-1}\right){\left(X_{k-1}-{\hat{X}}_{k-1}\right)}^{T}\right]{\Phi_{k,k-1}}^{T}=\\ E\left[K_{k}\nu_{k}{\nu_{k}}^{T}{K_{k}}^{T}\right]+E\left[\left(X_{k}-{\hat{X}}_{k}\right){\left(X_{k}-{\hat{X}}_{k}\right)}^{T}\right] \end{array}$$

The above formula can be simplified as:

(25) $${\hat{Q}}_{k}=K_{k}\nu_{k}\nu_{k}^{T}K_{k}^{T}+P_{k}-\Phi_{k,k-1}P_{k-1}\Phi_{k,k-1}^{T}$$

where $P_{k}$ is the predicted state covariance matrix at time *k*.

The formula is written in the form of a recursive estimation formula as:

(26) $${\hat{Q}}_{k}=(1-d_{k}){\hat{Q}}_{k-1}+d_{k}\left(K_{k}\nu_{k}\nu_{k}^{T}K_{k}^{T}+P_{k}-\Phi_{k,k-1}P_{k-1}\Phi_{k,k-1}^{T}\right)$$

Equation (26) is the recursive estimation formula of noise ${\hat{Q}}_{k}$ of the SHAKF algorithm.

**Figure 3:**
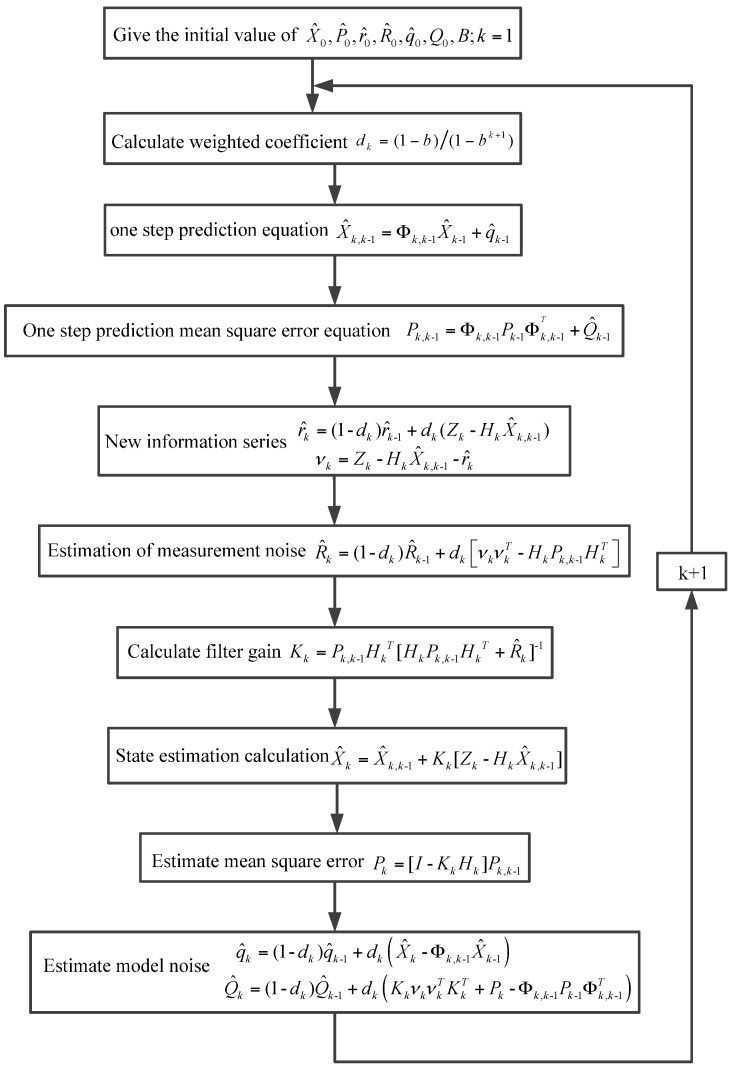
Flow chart of the SHAKF algorithm.

The flow chart of the SHAKF algorithm is shown in Figure 3.

As can be seen from Figure 3, the SHAKF algorithm consists of a noise estimator and a conventional Kalman filtering algorithm. The noise estimator can be used to estimate the real-time noise statistics, and the Kalman filtering algorithm can be adopted to complete the filtering state estimation.

#### 3.2.2. Analysis of the SHAKF Algorithm

The SHAKF algorithm is analyzed as follows:

1. The noise estimator cannot estimate the statistical properties of the process noise and measurement noise at the same time. Notice that the estimations of system noise and measurement noise both depend on the innovation, more specifically in the formula of $\nu_{k}\nu_{k}^{T}$. This is because $\nu_{k}\nu_{k}^{T}$ reflects the changes in the statistical characteristics of two kinds of noise at the same time, but in fact, $\nu_{k}\nu_{k}^{T}$ does not accurately reflect which kind of noise has changed. Assume that the measurement noise changes and process noise remain constant from a certain moment, then the covariance matrix of the measurement noise can be correctly estimated by means of Equation (22); and the estimated covariance matrix of the process noise obtained through Equation (26) is obviously inaccurate.
2. Suppose the expected value of measurement noise is ${\hat{r}}_{k}$, then the observation equation can be expressed as:
  (27) $$Z_{k}=H_{k}X_{k}+{\hat{r}}_{k}+V_{k}$$
  and ${\hat{r}}_{k}$ can be obtained as follows:
  (28) $${\hat{r}}_{k}=Z_{k}-H_{k}{\hat{X}}_{k}-V_{k}$$

It can be seen by comparison with Figure 3 that during the estimation of measurement noise expectation, the final estimated value ${\hat{X}}_{k}$ is substituted by a one-step prediction ${\hat{X}}_{k,k-1}$, so it is a kind of suboptimal algorithm.

Due to the fact the estimation of system process noise expectation and measurement noise expectation are suboptimal estimations, it may lead the expected estimation error to gradually accumulate and then produce large deviations during the recursive process and even interfere with the estimation of measurement noise or process noise variance.

3. The recursive formula of measurement noise covariance matrix $R_{k}$ is rewritten as follows:
  (29) $$\begin{array}{cc} {\hat{R}}_{k}= & \frac{b^{k}(1-b)}{1-b^{k+1}}{\hat{R}}_{0}+\frac{b^{k-1}(1-b)}{1-b^{k+1}}(\nu_{1}\nu_{1}^{T}-H_{1}P_{1,0}H_{1}^{T})+\frac{b^{k-2}(1-b)}{1-b^{k+1}}(\nu_{2}\nu_{2}^{T}-H_{2}P_{2,1}H_{2}^{T})+\\ & ...+\frac{1-b}{1-b^{k+1}}(\nu_{k}\nu_{k}^{T}-H_{k}P_{k,k-1}H_{k}^{T}) \end{array}$$

Notice that, in the estimation of $R_{k}$, the weight of the correction at the current time is maximum. Additionally, the weight gradually tends to a constant value of 1−b with the increase of time. Similarly, with the increase of k, the distribution weight, for which the initial value $R_{0}$ distributes on $R_{k}$, gradually decays and tends to zero. This shows that the adaptive degree of the estimator decreases gradually with the progress of filtering.

4. The recursive formula of $R_{k}$ can also be rewritten as follows:
  (30) $${\hat{R}}_{k}={\hat{R}}_{k-1}+\frac{1-b}{1-b^{k+1}}\left[\nu_{k}\nu_{k}^{T}-(H_{k}P_{k,k-1}H_{k}^{T}+{\hat{R}}_{k-1})\right]$$

It is known that:

(31) $$E[\nu_{k}\nu_{k}^{T}]=H_{k}P_{k,k-1}H_{k}^{T}+{\hat{R}}_{k}$$

On the one hand, it can be seen that during the recursive estimation of measurement noise, the autocovariance of the current measurement value is used. Actually, in the calculation of the autocovariance, ${\hat{R}}_{k}$ is substituted by ${\hat{R}}_{k-1}$, which confirms that the measurement noise estimator is a suboptimal noise estimator. On the other hand, the recursive formula requires the filter to tend to become stable, with the result that the noise estimation after calculating the difference makes the new estimation of the $R_{k}$ matrix lose the positive definite value. This is not because the estimation error variance matrix P is larger. In fact, the matrix P is generally chosen to be larger at the initial time of filtering, and a larger deviation can appear during the filtering process due to carrier maneuvers or innovation appearing, which causes coarse errors. When the estimated $R_{k}$ is negative definite because the matrix P is larger, it is likely to lead the filtering to diverge.

#### 3.2.3. Improvement of the SHAKF Algorithm

Aiming at the weight problem of the filtering algorithm and the divergence problem of the filter, we can think about treating the SHAKF algorithm as follows [24,25]:

1. A criterion of filtering convergence is introduced for the estimator, which is used to judge whether there is a large change in the measurement noise. The criterion is formulated as:
  (32) $$\nu_{k}^{T}\nu_{k}\leq \gamma^{2}Tr\left\{E\left[\nu_{k}\nu_{k}^{T}\right]\right\}$$
  where Tr(A) denotes the trace of the matrix A; γ is used to control the strictness degree of criterion, and its range is γ≥1. The specific method of use is: update the above criterion with new innovation; if the criterion is established, carry out the filtering; if not, indicating that the measurement noise variance changes greatly, then calculate the weight value $d_{k}$ from the initial value, i.e., the value of *k* is zero.

In the estimation of $R_{k}$, the degree of the utilization of the4 current innovation is maximum at the beginning of filtering. With the increase of time, $d_{k}$ is decreased; then, $R_{k}$ depends on $R_{k-1}$ much more than innovation. The tracking ability of measurement noise is improved by the above method.

2. The estimator of $R_{k}$ is rewritten in the following form:
  (33) $${\hat{R}}_{k}=(1-d_{k}){\hat{R}}_{k-1}+d_{k}\nu_{k}\nu_{k}^{T}$$
  where the related items of the one-step prediction error covariance matrix are cast out on the basis of Equation (22). Although the change is at the expense of certain filtering accuracy, the stability of the filter is improved.

## 4. Experiment and Analysis of Adaptive Filtering Based on the AR Model

### 4.1. The Filtering Equation

The offline analysis of static data in different environments from a certain type of FOG was developed by Casic33s by using the improved AR model and looks for the optimal fitting model. The results show that the improved AR(3) model can fit the FOG random drift well, so the improved AR(3) model is used to model online. The gyroscope precision can be described as follows: the constant error is 0.01°/h; the random drift error is $0.006^{{}^{\circ}}/\sqrt{h}$.

**Figure 4:**
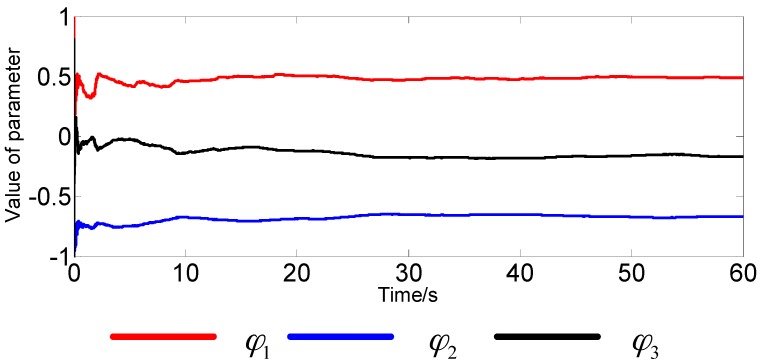
Real-time estimation of the parameter curve.

**Figure 5:**
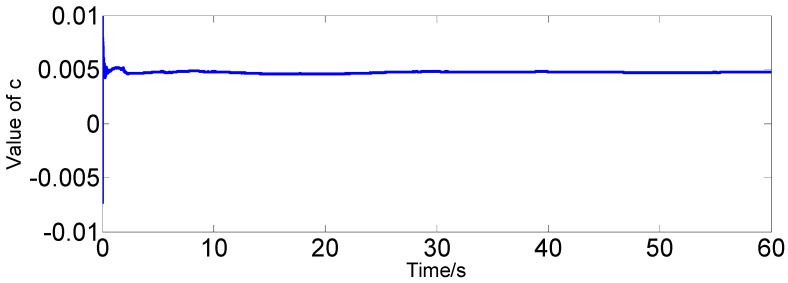
Real-time estimation of constant c.

Under static conditions, we record the real-time data of the FOG single axis (*x*-axis). According to Equation (4), the improved AR(3) model can be established online. Then, we adopt RLS to estimate the model parameters in real time. The real-time estimation of parameter curves is shown in Figure 4 and Figure 5. Finally, we carry out adaptive filtering on the FOG signal directly. As a result of using the FOG measured signal instead of the zero mean signal, the improved AR model has a constant *c*. In this paper, *c* is also considered as a state variable. Therefore, the system state equation can be expressed as follows:

(34) $$X_{k}=AX_{k-1}+BW_{k}$$

where state vector $X_{k}={\left[\begin{array}{cccc} z_{k} & z_{k-1} & z_{k-2} & c \end{array}\right]}_{1\times 4}^{T}$, process noise $W_{k}={\left[\begin{array}{cccc} a_{k} & 0 & 0 & 0 \end{array}\right]}_{1\times 4}^{T}$, $A={\left[\begin{array}{cccc} \varphi_{1} & \varphi_{2} & \varphi_{3} & 1\\ 1 & 0 & 0 & 0\\ 0 & 1 & 0 & 0\\ 0 & 0 & 0 & 1 \end{array}\right]}_{4\times 4}$, $B={\left[\begin{array}{cccc} 1 & 0 & 0 & 0\\ 0 & 0 & 0 & 0\\ 0 & 0 & 0 & 0\\ 0 & 0 & 0 & 0 \end{array}\right]}_{4\times 4}$.

Suppose the FOG output is $Z_{k}$, then the measurement equation of the system is as follows:

(35) $$Z_{k}=HX_{k}+V_{k}$$

where $H={\left[\begin{array}{cccc} 1 & 0 & 0 & 0 \end{array}\right]}_{1\times 4}$, $V_{k}$ is measurement noise.

As can be seen from the figures, in the absence of disturbance, the estimated parameters tend to become stable in about 30 s, and the estimated values are as follows:

(36) $$\varphi_{1}=0.4885,\;\varphi_{2}=-0.6660,\;\varphi_{3}=-0.1705,\;c=0.0048$$

Therefore, the improved AR(3) model is:

(37) $$z_{k}=0.4885z_{k-1}-0.6660z_{k-2}-0.1705z_{k-3}+0.0048+a_{k}$$

Then, the filtering equation is:

(38) $$\left\{ \begin{array}{l} \left[\begin{array}{c} z_{k}\\ z_{k-1}\\ z_{k-2}\\ c \end{array}\right]=\left[\begin{array}{cccc} 0.4885 & -0.6660 & -0.1705 & 1\\ 1 & 0 & 0 & 0\\ 0 & 1 & 0 & 0\\ 0 & 0 & 0 & 1 \end{array}\right]\left[\begin{array}{c} z_{k-1}\\ z_{k-2}\\ z_{k-3}\\ c \end{array}\right]+\left[\begin{array}{cccc} 1 & 0 & 0 & 0\\ 0 & 0 & 0 & 0\\ 0 & 0 & 0 & 0\\ 0 & 0 & 0 & 0 \end{array}\right]\left[\begin{array}{c} a_{k}\\ 0\\ 0\\ 0 \end{array}\right]\\ z_{k}=\left[\begin{array}{cccc} 1 & 0 & 0 & 0 \end{array}\right]\left[\begin{array}{c} z_{k-1}\\ z_{k-2}\\ z_{k-3}\\ c \end{array}\right]+V_{k} \end{array}\right.$$

According to the above filtering equation, the random drift error of FOG can be filtered using SHAKF.

### 4.2. Static Experiment Results and Analysis

**Figure 6:**
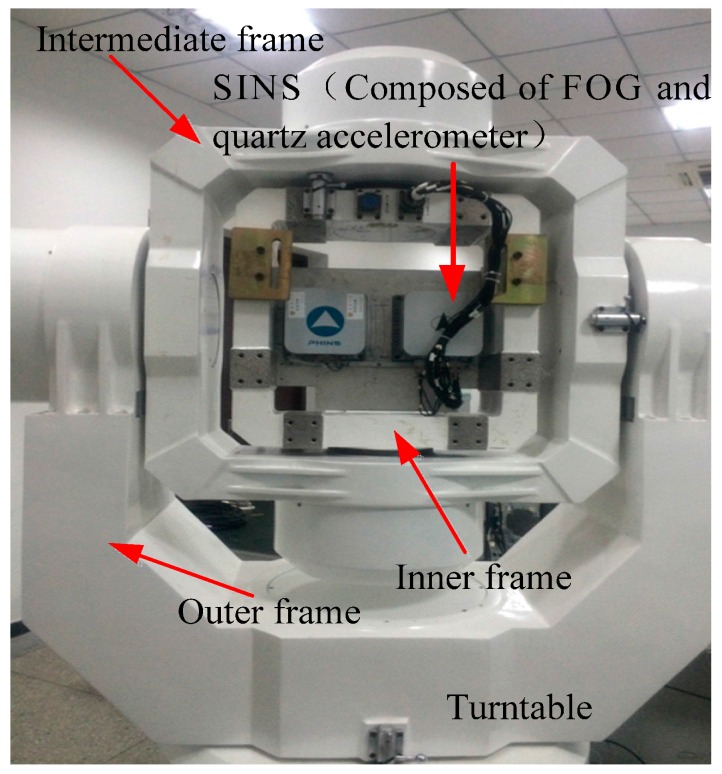
Experimental setup of FOG.

Under static conditions, we record the real-time data of a single axis (*x*-axis) FOG for 2 h. The sampling period is 5 ms, and the experimental picture is shown in Figure 6. 

**Figure 7:**
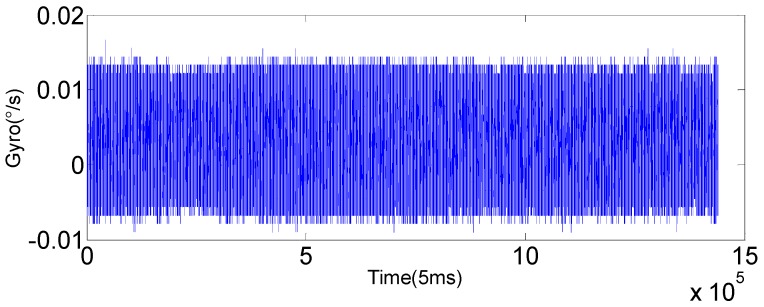
Raw output of the gyroscope (*x*-axis).

**Figure 8:**
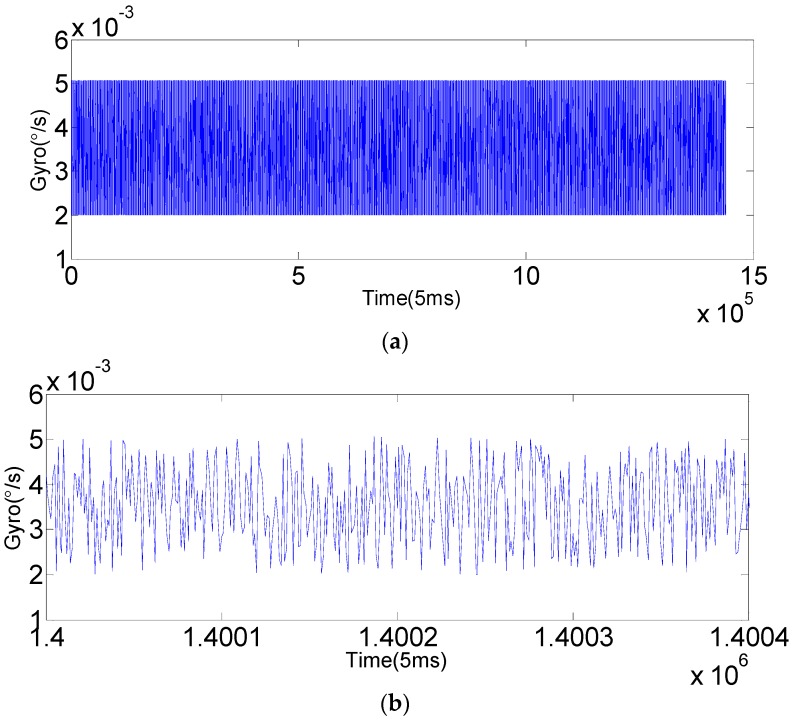
The filtering result of KF. (**a**) The filtering result of a single axis (*x*-axis) FOG for 2 h; (**b**) local enlarged graphs.

**Figure 9:**
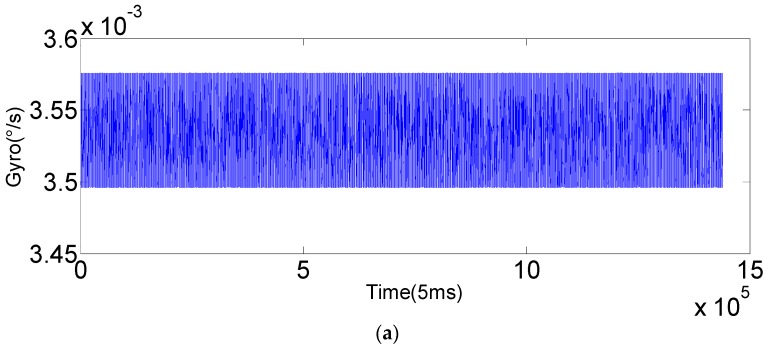

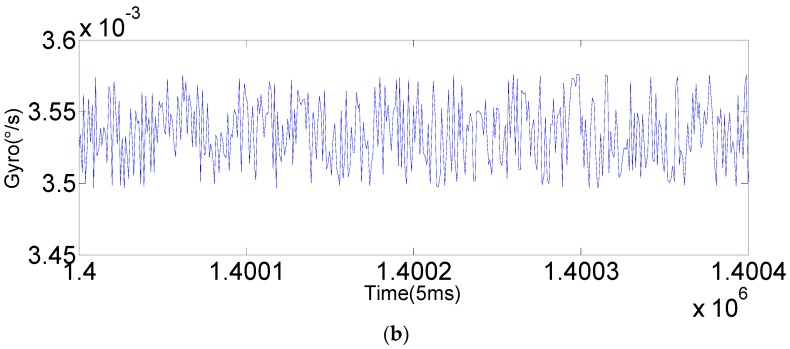
The filtering result of the modified SHAKF. (**a**) The filtering result of a single axis (*x*-axis) FOG for 2 h; (**b**) local enlarged graphs.

The original output of gyroscope (*x*-axis) is shown in Figure 7, and the results with KF and SHAKF processing are shown in Figure 8 and Figure 9. 

As can be seen from the figures, after filtering by the SHAKF, the noise is significantly reduced, while the average value remains unchanged. The filtering effect of SHAKF is better than that of KF. According to the filtering results, on the one hand, it can be seen that using the improved AR model of FOG to model can effectively reduce the gyroscope random drift error; the validity of the denoising method is verified. On the other hand, the effect of using the KF is not as good as the SHAKF, which indicates that when dealing with the filtering problem of an uncertain model, the adaptive filtering algorithm has better robustness.

#### Allan Variance Analysis

Now, the sampling data, the data of the random drift model and the data after filtering by the KF and SHAKF are compared by adopting the Allan variance analysis method. The Allan variance method is a time domain analysis method, which is recognized as a standard method for the analysis of FOG parameters by IEEE [26]. It can characterize and identify all kinds of error sources and their contribution to the statistical properties of the whole noise very easily and meticulously. Therefore, the Allan variance analysis can be used to analyze the variation of error coefficients before and after filtering FOG signals quantitatively. If the noise sources are independent of each other, then the Allan variance is the sum of squares of each type of error. The Allan variance of FOG can be expressed as [27,28,29,30,31]:

(39) $$\sigma_{total}^{2}(\tau )=\sigma_{ARW}^{2}(\tau )+\sigma_{BI}^{2}(\tau )+\sigma_{RRW}^{2}(\tau )+\sigma_{RR}^{2}(\tau )+\sigma_{QN}^{2}(\tau )$$

Namely,

(40) $$\sigma_{\Omega }^{2}(\tau )=\frac{R^{2}}{2}\tau^{2}+\frac{K^{2}}{3}\tau +\frac{2B^{2}\ln2}{\pi }+N^{2}\tau^{-1}+3Q^{2}\tau^{-2}$$

where ARW is the angle random walk, and its error coefficient is N; BI is the bias instability, and its error coefficient is B; RRW is the rate random walk, and its error coefficient is K; RR is the rate ramp, and its error coefficient is R; QN is the quantization noise, and its error coefficient is Q.

According to Equation (35), if we use the least square method to fit the data, we can get the error coefficients of five noise sources before and after filtering the FOG signal. They are shown in Table 1.

It can be seen from Table 1 that the sampling data are basically consistent with the noise characteristics of the noise sequence fitted by the improved AR model. The fitting accuracy of the rate ramp and rate random walk error are both more than 99%. Due to the fact that the magnitudes of the angle random walk, bias instability and quantization noise are small, fitting is more difficult, but the fitting accuracy reaches above 93%. After the data are processed by two filtering methods, the five noise source error coefficients of the FOG output signal are obviously reduced. After adopting the modified SHAKF, the noise source error coefficients are all less than half of their values before filtering; for example, the FOG bias stability reduces from the original 0.0007122 (°/h) to 0.0003523 (°/h). When analyzing the filtering effect of a single noise, the modified SHAKF can improve the performance by up to 42.5% (quantization noise) compared to KF, and the filtering effects of the random walk and rate ramp error are increased by 34.1% and 38.5%, respectively. It can be seen that the effect of the modified SHAKF is better than KF. Therefore, the random drift error of FOG is effectively reduced, and the accuracy of FOG is improved.

### 4.3. Dynamic Experiment Results and Analysis

In order to verify the applicability of the modified model under moving base conditions, the gyroscope axis (*x*-axis) is rotated by 5°/s, 15°/s, 25°/s, 35°/s and 50°/s, respectively. In this paper, the dynamic data of FOG (*x*-axis) is recorded for 2 h at room temperature with a sampling period of 5 ms. The improved AR(3) model is established as the system state equation using 1,440,000 differentiated samples. Then, the SHAKF algorithm is applied to denoise the dynamic signal of the FOG with the same initial parameters chosen as under static conditions. The results are shown in the following figures, where the blue curve represents the FOG noisy signal, the red curve indicates the SHAKF denoising signal and the green curve denotes the KF denoising signal.

**Figure 10:**
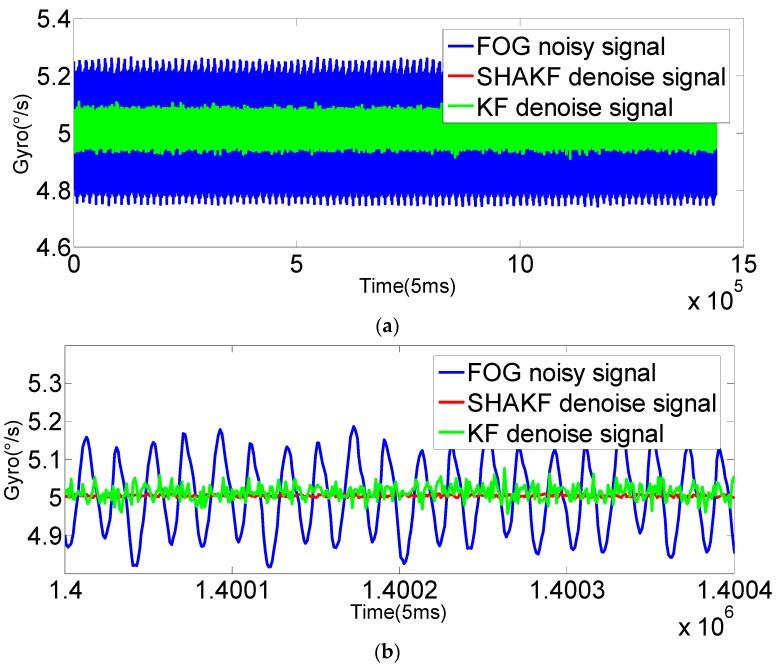
Comparison of the results before and after denoising for the FOG dynamic signal at a rate of 5°/s. (**a**) Denoising the results of the FOG dynamic signal (*x*-axis) for 2 h; (**b**) local enlarged graphs.

**Figure 11:**
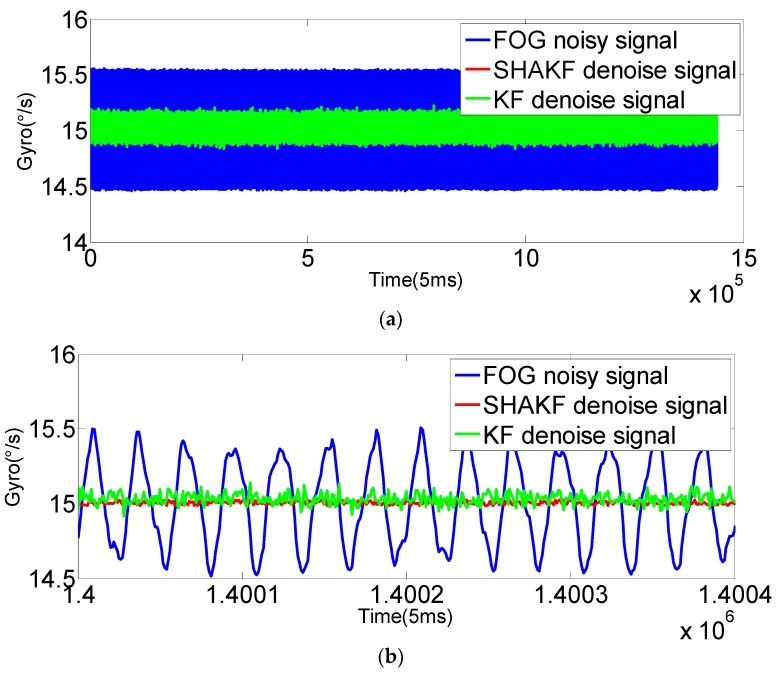
Comparison of the results before and after denoising for the FOG dynamic signal at a rate of 15°/s. (**a**) Denoising the results of the FOG dynamic signal (*x*-axis) for 2 h; (**b**) local enlarged graphs.

**Figure 12:**
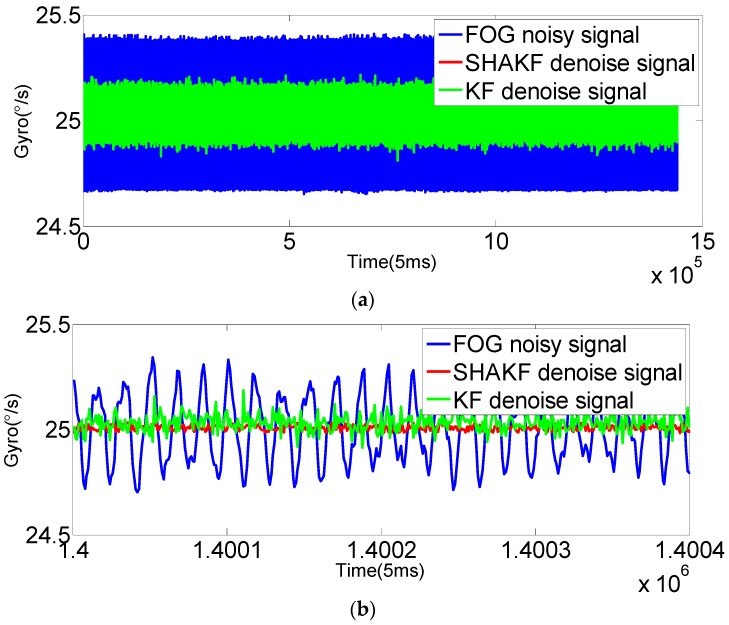
Comparison of the results before and after denoising for the FOG dynamic signal at a rate of 25°/s. (**a**) Denoising results of the FOG dynamic signal (*x*-axis) for 2 h; (**b**) local enlarged graphs.

**Figure 13:**
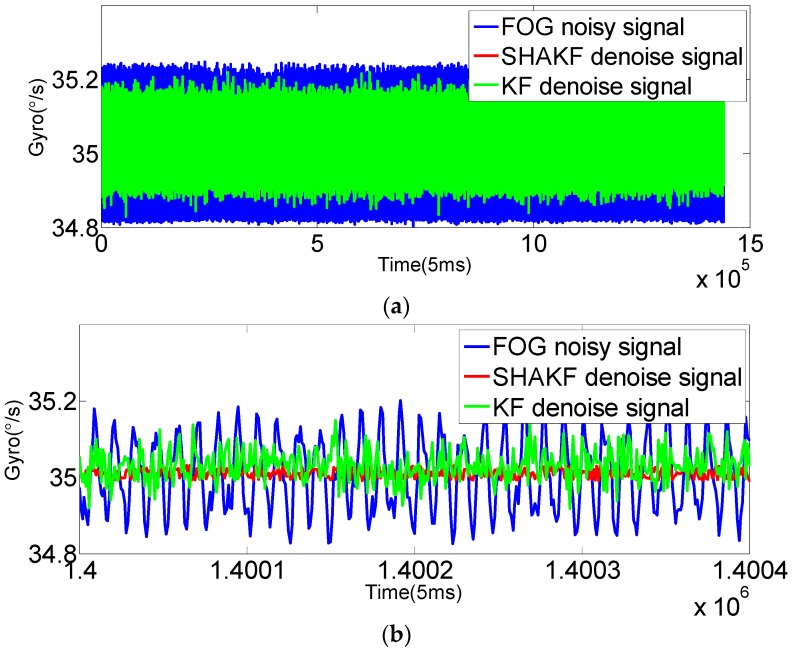
Comparison of the results before and after denoising for the FOG dynamic signal at a rate of 35°/s. (**a**) Denoising results of the FOG dynamic signal (*x*-axis) for 2 h; (**b**) local enlarged graphs.

**Figure 14:**
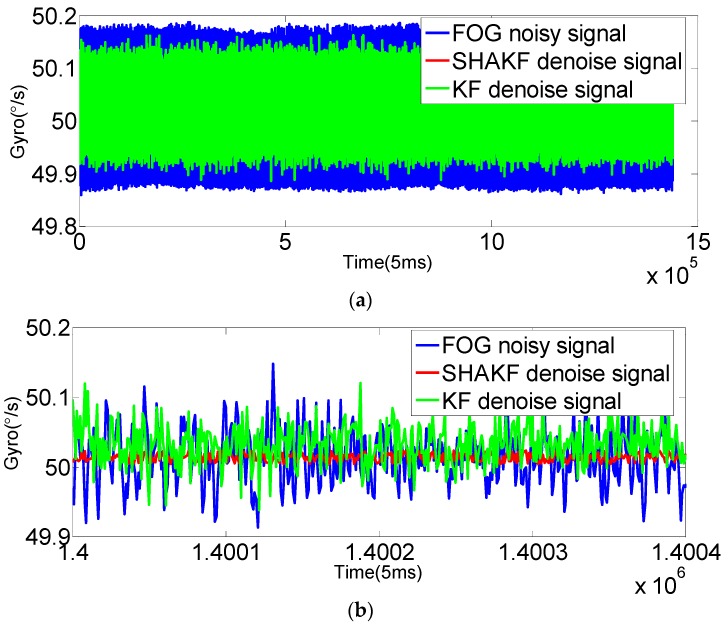
Comparison of the results before and after denoising for the FOG dynamic signal at a rate of 50°/s. (**a**) Denoising results of the FOG dynamic signal (*x*-axis) for 2 h; (**b**) local enlarged graphs.

It can be seen from Figure 10, Figure 11, Figure 12, Figure 13 and Figure 14 that the denoising effect of the KF becomes worse and worse with the increasing of rotational velocity, even becoming invalid. Relative to the KF, the denoising effect of the modified SHAKF is very obvious, and any increase in rotational velocity has little influence on SHAKF.

Mean square error (MSE), root mean square error (RMSE) or the signal-to-noise power ratio (SNR) [9] are generally employed to compare the performance of denoising methods before and after denoising the FOG dynamic drift signal. The MSE is defined as follows:

(41) $$MSE=\sqrt{\frac{1}{N}{\sum_{t=1}^{N}(x(t)-\bar{x}(t))}^{2}}$$

where $\bar{x}(t)$ is the mean value of the signal, x(t) is the actual signal and N is the number of signals. The MSE results calculated before and after denoising are in Table 2.

Through comparing the statistical characteristics of the FOG output signal before and after denoising, it can be seen that the MSE of random drift error before and after filtering clearly changes. Additionally, the MSE of SHAKF denoised signal is much less than that of KF. Therefore, it can be concluded through the above analysis that the modified SHAKF is a suitable denoising algorithm for reducing the FOG random drift error.

## 5. Conclusions

In this paper, the improved AR(3) model is proposed. It is shown that a model of the FOG output signal can be established online by directly using the output static data of a FOG. Additionally, according to the model, the modified SHAKF is adopted to filter the FOG random drift errors in real time, which effectively reduces FOG errors and improves the accuracy of FOG. Static experiments and dynamic experiments were done to verify the effectiveness of this method. Under static conditions, the filtering performance of the modified SHAKF is compared to KF, and it is proven to be superior to KF. Based on Allan variance analysis, the random errors, like the angle random walk and bias instability, are reduced two-fold. The fitting precision of the FOG random drift model established online by the improved AR model is higher. The real-time performance is strong, and the minimum fitting accuracy of single noise is 93.2%. Under dynamic conditions, the minimum MSE obtained by the modified SHAKF shows that the improved AR model established under static conditions is also perfect. The effectiveness of this method is validated in denoising the single-axis FOG signal under both static and dynamic conditions. The research presented in this paper is of great significance in engineering applications.

## Figures and Tables

**Table 1:** Comparison of the noise source error coefficients before and after filtering.

Error Coefficients (Unit)	Original Signal	Fitting Sequence	Kalman Filtering	SHAKF
$N({}^{\circ}/h^{1/2})$	4.2026 × 10^−6^	3.9168 × 10^−6^	3.2547 × 10^−6^	2.0632 × 10^−6^
B(°/h)	7.1221 × 10^−4^	7.0438 × 10^−4^	6.1221 × 10^−4^	3.5235 × 10^−4^
$K({}^{\circ}/h^{3/2})$	0.0335	0.0334	0.0246	0.0162
$R({}^{\circ}/h^{2})$	0.5401	0.5390	0.4286	0.2636
Q(°)	1.1565 × 10^−5^	1.1195 × 10^−5^	9.4795 × 10^−6^	5.4532 × 10^−6^

**Table 2:** MSE results of the FOG signal with different rotational velocities.

Rotation (°/s)	FOG Signal (°/s)	KF Denoised Signal (°/s)	SHAKF Denoised Signal (°/s)
5	0.1068	0.0362	0.0184
15	0.2461	0.0932	0.0396
25	0.1629	0.1086	0.0280
35	0.0844	0.0637	0.0145
50	0.0538	0.0445	0.0093
